# Nanog induced intermediate state in regulating stem cell differentiation and reprogramming

**DOI:** 10.1186/s12918-018-0552-3

**Published:** 2018-02-27

**Authors:** Peijia Yu, Qing Nie, Chao Tang, Lei Zhang

**Affiliations:** 1 Center for Quantitative Biology, Peking University, Beijing, 100871 China; 2 Department of Mathematics and Departmentof Developmental and Cell Biology, University of California Irvine, Irvine, CA 92697 USA; 3 Peking-Tsinghua Center for Life Sciences, Peking University, Beijing, 100871 China; 4 Beijing International Center for Mathematical Research, Peking University, Beijing, 100871 China

**Keywords:** Gene network, Stem cells, Cell differentiation, iPS cell reprogramming, Intermediate cellular state, Nanog

## Abstract

**Background:**

Heterogeneous gene expressions of cells are widely observed in self-renewing pluripotent stem cells, suggesting possible coexistence of multiple cellular states with distinct characteristics. Though the elements regulating cellular states have been identified, the underlying dynamic mechanisms and the significance of such cellular heterogeneity remain elusive.

**Results:**

We present a gene regulatory network model to investigate the bimodal Nanog distribution in stem cells. Our model reveals a novel role of dynamic conversion between the cellular states of high and low Nanog levels. Model simulations demonstrate that the low-Nanog state benefits cell differentiation through serving as an intermediate state to reduce the barrier of transition. Interestingly, the existence of low-Nanog state dynamically slows down the reprogramming process, and additional Nanog activation is found to be essential to quickly attaining the fully reprogrammed cell state.

**Conclusions:**

Nanog has been recognized as a critical pluripotency gene in stem cell regulation. Our modeling results quantitatively show a dual role of Nanog during stem cell differentiation and reprogramming, and the importance of the intermediate state during cell state transitions. Our approach offers a general method for analyzing key regulatory factors controlling cell differentiation and reprogramming.

**Electronic supplementary material:**

The online version of this article (10.1186/s12918-018-0552-3) contains supplementary material, which is available to authorized users.

## Background

Phenotypic cell-to-cell heterogeneity has been viewed as an important hallmark for both pluripotent embryonic stem (ES) cells [1–4] and multipotent adult stem cells [5–7]. Such non-genetic heterogeneity within stem cell populations suggests the co-existence of multiple cellular states manifested by transcriptome-wide gene expression patterns [2, 5]. The dynamical interconversion among these gene expression states is driven by various types of fluctuations, including protein and RNA (mRNA, miRNA) expression noise in gene regulatory networks for cell fate determination [8–10], the noise of external input signals [11], the re-establishment of histone modification pattern on the chromatins of daughter cells after each cell division [12].

Nanog has been widely studied as one of the heterogeneously expressed genes in ES and induced pluripotent stem (iPS) cell populations [13, 14]. It plays a central role in maintaining the cellular self-renewal and pluripotency [15–17]. In terms of the gene regulatory network defining cellular potency, Nanog has direct mutual interactions with two other core stem cell specific genes Oct4 and Sox2. They occupy the core position of the network, and link up most downstream genes controlling cellular stemness and cell fate specification [18–21].

Experimental data from mice ES cell lines indicate that the Nanog expression level in cell population shows a typical bimodal distribution: Approximately 80% cells show a relatively high level of Nanog, while the rest of 20% cells keep a low level of Nanog which is close to the Nanog expression level in differentiated cells [22–24]. This can be partly explained by cell differentiation due to some environmental factors in the experiments [22, 24]. However, after sorting and purifying multiple populations of ES cells with different Nanog expression levels, and the subsequent self-renewal culture of these “sub-populations”, the same bimodal Nanog distribution can be eventually re-established from all sub-populations with the scale of recovering time for about 10 days [22, 24]. These results suggest the co-existence of two cellular “sub-states” within the pluripotent stem cell state: high-Nanog sub-state and low-Nanog sub-state, rather than one well-defined, homogenous pluripotent cellular state. Furthermore, the two sub-states spontaneously go through reversible interconversion toward a dynamical equilibrium.

Very few studies have addressed the functional roles of the bimodal heterogeneity of Nanog expression in the differentiation and specification process of stem cells. Previous experimental results have shown that the low-Nanog stem cell sub-populations are more likely to differentiate than high-Nanog sub-populations, under the induction of differentiation signals [24–27]. One hypothesis based on these results is: During the stem cell differentiation process, the low-Nanog state of stem cell functions as the “gate-keeper” state. The preparation of the “gate-keeper” state of stem cells and the presence of external signals are two necessary conditions for stem cell differentiation [10, 26, 27].

Some previous modeling works have attempted to study the Nanog bimodal distribution and the dynamical interconversion between high/low-Nanog states based on network topology and gene regulatory interactions, including stochastic transition between the two states driven by expression noise [25] and gene expression oscillation [28, 29]. For instance, Glauche et al. modeled a three-node network including Oct4, Nanog and one unknown Nanog repressor based on bistable switch mechanism or oscillatory mechanism [25]. The stochastic bistable model often requires a high level of gene expression noise to fit the variation observed in the experiment, and lacks the ability to explain different variation levels for different genes. On the contrary, the excitable model can have relatively large excitable excursions in the phase space triggered by much smaller noise to avoid such disadvantages. Excitation mechanism has been utilized to explain bimodal distribution of gene expression and stochastic transitions among multiple metastable cell fates [30–32]. In particular, Kalmar et al. proposed a simplified two-dimensional Oct4-Nanog network based on excitation mechanism to model bimodal distribution of Nanog [24]. However, one limitation of the previous models for Nanog heterogeneity is that all lineage specification genes are neglected, thus the role of Nanog stochasticity in stem cell differentiation and iPS cell reprogramming cannot be quantitatively analyzed.

Here, we propose a mathematical model to investigate the contrary roles of Nanog in stem cell differentiation and iPS cell reprogramming. By introducing a five-node stem cell gene regulatory network, we first verify the Nanog bimodal distribution and high/low-Nanog state interconversion, and then investigate the functional roles of Nanog bimodality. For the role of Nanog in stem cell differentiation, we show that the low-Nanog state serves as an intermediate state in the differentiation process, which is a stricter description than the “gate-keeper state”. The differentiation process through the low-Nanog intermediate has a lower barrier on the potential landscape so that it is more prone to happen. While for the iPS cell reprogramming, we demonstrate the existence of low-Nanog state dynamically leads to a negative effect, and additional Nanog activation is necessary for increasing reprogramming efficiency via preventing reprogrammed iPS cell from trapping in the low-Nanog state by over-activated Oct4/Sox2.

## Results

### The five-node stochastic gene regulatory network controlling cellular stemness

**Fig. 1:**
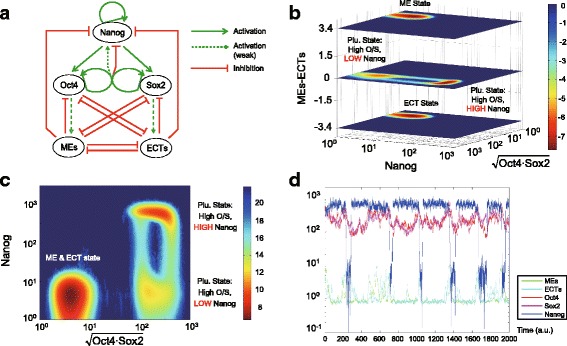
The stochastic gene regulatory model of cell fate determination with Nanog and lineage specifiers. **a** The five-node gene regulatory network of cell fate determination. The network includes three stemness genes: Oct4, Sox2, Nanog, and two mutual antagonistic lineage specifiers: MEs and ECTs. **b** and **c** The cell-state landscape of multiple cell attractors, produced from trajectory density sampling. The red-color regions indicate relatively high sampling probability, corresponding to low value of − log(*P*_*ss*_) on potential landscape. **b** The five-dimensional phase space is transformed to three-dimensional one, where the x-axis represents the geometric average of Oct4 and Sox2, the y-axis for Nanog, and the z-axis indicates the difference between MEs and ECTs. **c** The landscape in a two-dimensional phase plane, where the MEs-ECTs axis is compressed. **d** Typical temporal trajectories of stochastic gene expression of stem cell state. Frequent dynamical transitions between high- and low-Nanog states can be seen (blue line)

We first build a five-node gene regulatory network model, including three pluripotent genes: Oct4, Sox2, Nanog, and two mutually antagonistic lineage specifiers: MEs (representing mesendoderm genes) and ECTs (representing ectoderm genes) (Fig. 1a). The modeling of interactions among Oct4, Sox2, MEs and ECTs is inherited from the previous four-node “Seesaw” model for the iPS cell reprogramming [33], including self-activation of Oct4 and Sox2, mutual repression between MEs and ECTs, and multiple interactions among pluripotent genes and specifier genes. Both Oct4 & Sox2 and MEs & ECTs are assumed to be symmetric for simplicity. In the five-node network, we introduce an additional stem cell gene: Nanog. The regulations among Oct4, Sox2 and Nanog consist of self-activation of Nanog, activation of Nanog to Oct4 and Sox2 [18, 34], and one negative feedback of combined Oct4-Sox2 to Nanog. For the negative feedback, low concentration of Oct4-Sox2 weakly activates Nanog, while high concentration of Oct4-Sox2 strongly represses Nanog, which is supported by some experimental evidence [18, 34]. Stochastic differential equations are used to formulate this five-node network (see Methods and Materials for model details and Additional file 1: Table S1 for the choice of parameters).

### Stochastic transition between high-Nanog state and low-Nanog state

We performed simulations of the proposed model to examine the dynamics of Nanog gene expression. The computational results of trajectory density sampling in the phase space produced four regions with relatively high sampling probability, implying stable or metastable cellular states (Fig. 1b and c). Two of them represent the differentiated cellular states: ME state & ECT state, where one of the MEs or ECTs gene groups is highly expressed, and Oct4, Sox2, Nanog are low expressed for both two states (Additional file 2: Figure S1). The other two states correspond to the high-Nanog and low-Nanog cell subpopulations within the heterogeneous pluripotent cellular state (both of them with high Oct4/Sox2 level) (Fig. 1d). The negative feedback loop between Nanog and Oct4/Sox2 plus the weak activation from Oct/Sox2 to Nanog constitute an excitable system, leading to a recurring stochastic transition between high-Nanog and low-Nanog sub-states even with relatively low gene expression noise (Fig. 1d). In order to reveal the excitable mechanism in a more generalizable way, we further abstracted a minimal simplified two-dimensional Oct4-Nanog model and preformed the phase plane analysis as illustrated in Additional file 3: Figure S2A (see the formulation in Methods and Materials and the parameters in Additional file 4: Table S2).

**Fig. 2:**
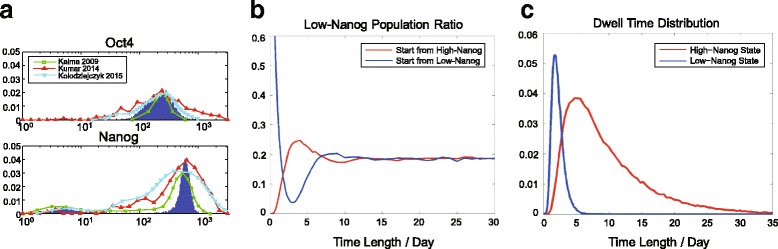
Bimodal gene expression distribution and stochastic transitions between states with high / low Nanog level of the model. **a** Distributions of Oct4 and Nanog level within simulated cell population (*N* = 10,000) (blue lines). Oct4 shows a single-peak distribution (μ=236.1, σ=111.8), and Nanog a bimodal distribution. The fraction of low-Nanog population is 18.9%. (Low-Nanog population, μ=6.0, σ=4.4; high-Nanog population, μ=584.2, σ=130.0.) For comparison, the green squares are the experimental flow cytometry data sets of Oct4 and Nanog from Kalmar et al. 2009 [24], the red triangles correspond to the single-cell RNA-seq data in Kumar et al. 2014 [4], and the blue triangles correspond to the single-cell RNA-seq data in Kolodziejczyk et at 2015 [35]. **b** Simulations of the re-establishment toward dynamical equilibrium of high/low-Nanog states. For simulated cell population (*N* = 10,000) with the initial high-Nanog condition (red curve) and the low-Nanog condition (blue curve), the fraction of low-Nanog subpopulation is tracked in the course of time, and it can eventually recover to ~ 20% for both two groups after ~ 10 days. **c** Distribution of dwell time of high/low-Nanog sub-states within the pluripotent stem cell state

The bimodal distribution of Nanog expression level within the pluripotent cell population under dynamical equilibrium can be naturally generated from our five-node model (Fig. 2a and Additional file 3: Figure S2B), which is able to quantitatively fit the experimental data better than the original fitting by the Kalmar’s model [24]. Also, the simulation qualitatively matches the normalized results from two single cell RNA-seq data sets (using mouse ES cells cultured in serum+LIF) [4, 35], which show a high-level expression of Oct4 and a bimodal expression of Nanog in Fig. 2a, but a broader distribution of the Sox2 expression compared to the simulation. To verify that the distribution of high/low-Nanog cell sub-populations are under interconversion toward dynamical equilibrium, we repeat the re-establishment process of Nanog expression bimodality from sorted stem cell sub-populations, initiating from all high-Nanog state cells, or all low-Nanog state cells. Both two cell sub-population groups could re-establish the Nanog bimodal distribution after reaching the dynamical equilibrium for ~ 10 days [18, 34], displayed in Fig. 2b. The distributions of the dynamical dwell time at the high/low-Nanog states are shown in Fig. 2c. Note that they do not obey exponential distribution, which is one characteristic of stochastic transition between bistable switch systems, indicating an essential difference of excitable system and stochastic bistable switch.

### Nanog as “catalyst”: Differentiation through the low-Nanog state has a lower energy barrier

Now we consider possible functional roles of Nanog dynamics in the process of stem cell differentiation. In order to investigate the functional role of Nanog dynamics in the five-node network model, it is necessary to introduce a control model that excludes the dynamics of Nanog. To build up the control model, the concentration value of Nanog in the five-node model is fixed as a constant, and the steady state values of the other four genes remain unchanged (see details of the control model in Methods and Materials). Therefore, we are able to compare the parallel behaviors of the wild-type (WT) model (with Nanog dynamics) and the control model (without Nanog dynamics).

In order to quantitatively assess the tendency of cell state transition from the stem cell state (the high-Nanog sub-state in the WT model, or the homogeneous pluripotent state in the control model) toward one of the differentiated states driven by gene expression noise (rather than by parameter changing and bifurcation), we employ the minimum action path (MAP) to locate the transition path based on the Wentzell-Freidlin large deviation theory [36]. This approach allows us to calculate the most probable transition path between the initial and final states in a random dynamic system by minimizing the action functional, and has been successfully applied in many biological systems, such as gene switching in zebrafish hindbrain [37] and budding yeast cell cycle [38].

By calculating the transition paths (i.e., MAPs) of cellular differentiation for the WT model and the control model, the quantitative comparison of differentiation tendency is proceeded by two ways: (i) The value of the potential landscape along the transition path; (ii) The monotonous increasing action function along the transition path. For the first approach, we apply the landscape function for complex biological network dynamics, which often corresponds to the non-equilibrium systems, to serve as an analogy of the energy function in the equilibrium systems. There are three representative landscape theories in literatures: 1) the “potential landscape” from the steady state distribution of stochastic differential equations (SDEs) [39]; 2) the “quasi-potential” from Freidlin-Wentzell’s large deviation theory [38]; 3) the potential function obtained through the SDE decomposition by the fluctuation-dissipation theorem [40], which can be viewed as a mapping between a non-equilibrium dynamical system and a Hamiltonian system [41]. Here, we use the potential landscape defined by the negative logarithm of the steady state probability distribution *P*_*ss*_ as the landscape function, i.e., − log(*P*_*ss*_), which is the same one used in [39].

The potential landscape along the transition path is similar to the free energy along the reaction coordinates, and it is very intuitive to illustrate the transition intermediates and the energy barriers. However, it should be noticed that the potential landscapes only provide rough sketches because the gene regulatory network systems are usually far from gradient systems. The transition path could be different from the reaction coordinates. Thus, we also apply the second approach to calculate the action function along the transition path for further validation. The action barrier is a much stricter physical quantity than the energy barrier to describe the transition tendency and estimate the transition rate [36].

**Fig. 3:**
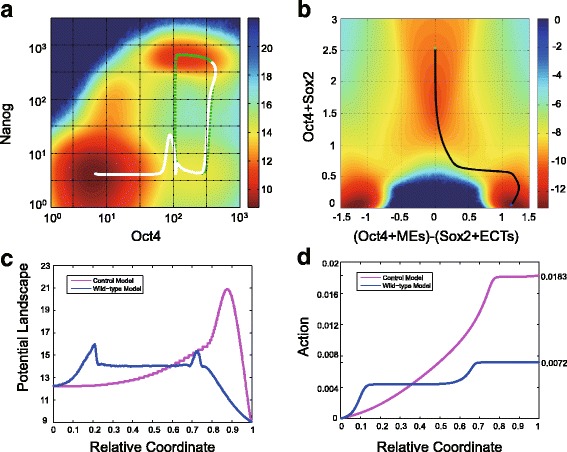
The low-Nanog state enhances cell differentiation through lower the transition barrier. **a** The potential landscape (computed by the trajectory density sampling) for the wild-type (WT) model. The white curve indicates the minimum action path (MAP) from the pluripotent state (green dot) to the ME state (blue dot). The green dotted line is a typical ODE trajectory showing the excursion due to the excitability of the system. **b** The control model (excluding the dynamics of Nanog). The black curve is the MAP from the pluripotent state (green dot) to the ME state (blue dot). **c** and **d** Comparison between the WT model (blue) and the control model (purple). The value of the potential landscape (**c**) and the action value (**d**) along the relative coordinate ([0,1]) of the MAP from the pluripotent state to the ME state

We apply the minimum action method in [42] to compute the minimum action path of differentiation for the WT model (Fig. 3a) and the control model (Fig. 3b) (see numerical method in Methods and Materials). For the WT model, the most probable path (i.e., minimum action path) passes the low-Nanog sub-state on the potential landscape, which is robust to the selection of initial path for the action minimization algorithm (Additional file 5: Figure S4). Also, it superimposes the first half of the excitable excursion of the typical ODE trajectory illustrated in green dotted line in Fig. 3a, showing the connection between the system excitability and the differentiation transition path. In the WT model, the energy along the transition path is a typical double-peak plot (blue curve in Fig. 3c), and the action is a typical two-stage stepwise function (blue curve in Fig. 3d), indicating the existence of one intermediate and two barriers along the transition path. The corresponding low-Nanog sub-state on the landscape (Fig. 3a), the intermediate state of the potential landscape (blue curve in Fig. 3c), and the plateau part of the monotonous action (blue curve in Fig. 3d) all point to the same conclusion: the low-Nanog state serves as the intermediate state of the differentiation process. For the transition path in the control model, the potential landscape (pink curve in Fig. 3c) has a single large peak, and the action is one-stage stepwise (pink curve in Fig. 3d), indicating there is no intermediate state for the differentiation state transition process in the control model.

By comparing both barriers of potential landscape (Fig. 3c) and action values (Fig. 3d) of the entire transition path of the WT model and the control model, we arrive at an important conclusion that the existence of the low-Nanog intermediate state during the differentiation process can significantly decrease the barrier of potential landscape and the action value along the transition path. This can be also understood qualitatively: low Nanog expression level leads to temporary low level of Oct4 and Sox2, which in turn release the inhibition of Nanog to the lineage specifier genes, making the high expression of one specifier gene and the differentiation process more prone to happen. Such a role of low-Nanog intermediate state is intuitively similar to the enzyme in catalysis process. The enzyme-substrate complex is often an intermediate state between the substrate and the product that significantly decreases the energy barrier of the chemical reaction.

### Intrinsic bimodal Nanog expression slows down iPS reprogramming, while exogenous induction of Nanog improves reprogramming efficiency

Next we investigate how bimodal heterogeneity of Nanog may affect induced cellular reprogramming process, i.e., the enforced transition process from somatic cell state (ME or ECT state) to the stem cell state. First, we notice that the minimum action path of reprogramming without additional exogenous Nanog induction does not pass through the Nanog intermediate state, very different from the path of differentiation (Additional file 6: Figure S5).

A common way of iPS cell reprogramming is to activate, or repress the expression of particular gene groups with various types of external inductions, such as virus transformation of vectors expressing target gene products, RNA interference, and small chemical molecule stimulation [13, 14]. Activating gene inductions can be modeled as constant terms indicating additional basal expression rates, noted as (*C*_*O*_, *C*_*S*_, *C*_*N*_, *C*_*M*_, *C*_*E*_) below, and repressive inductions can be represented by multipliers of regulated production rate, noted as (*I*_*O*_, *I*_*S*_, *I*_*N*_, *I*_*M*_, *I*_*E*_). Details of the mathematical formulations are described by Eqs. (3.1–3.5) in Methods and Materials.

One may adjust the induction parameters in different combinations to change the Waddington landscape of iPS induction to enable the induced somatic cells falling into the basin for stem the cell. At the end of the induction process, the induction parameters are re-set to the original values, in order to model the retraction or degradation of the input vectors, RNA or other chemical molecules [43]. The original Waddington landscapes will be then restored and the successfully induced cells will be permanently switched to the stem cell state.

We notice that the pluripotent cellular state always splits up into two sub-states with high/low Nanog level due to spontaneous dynamical transition, i.e., all successfully reprogrammed cells have the heterogeneous stem cell state, with ~ 80% in high Nanog level and ~ 20% in low. However, the cells with high Oct4, Sox2 and low Nanog expression are usually regarded as the incomplete reprogrammed cells, as suggested by previous work that the high Nanog expression could activate the related downstream genes in the late stages of cell reprogramming process, to promote the incomplete reprogrammed cells towards the full pluripotent state [16, 17]. The time period of reprogramming usually lasts for tens of weeks [43]. If Nanog maintains a low expression level during the long induction process, the expression of other downstream genes of Nanog will remain repressed, leading to longer time to attain full pluripotency. Thus, the long-time existence of low Nanog level will increase the time of reprogramming [16, 17].

**Fig. 4:**
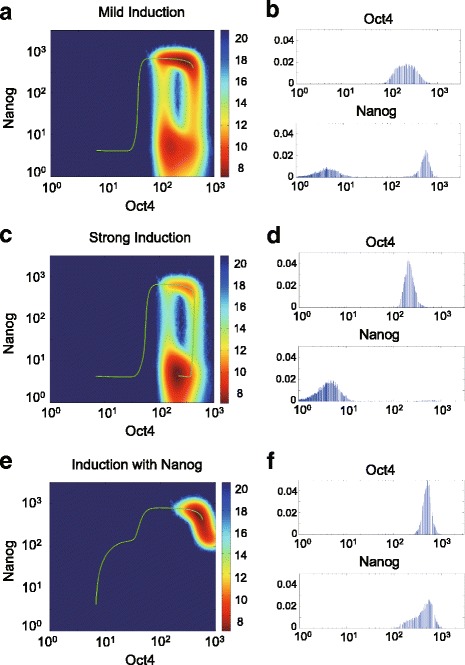
The Nanog expression level during induction with different strategies of iPS cell reprogramming. **a**, **c**, **e** Three reprogramming strategies of activating Oct4 and Sox2. **a** mild induction (*C*_0_ = *C*_*S*_ = 0.1): the high-Nanog subpopulation still makes up the majority; (**c**) strong induction (*C*_0_ = *C*_*S*_ = 0.3): the low-Nanog subpopulation becomes the majority and reduces the reprogramming efficiency; (**e**) strong induction with additional Nanog activation (*C*_0_ = *C*_*S*_ = *C*_*N*_ = 0.3): the low-Nanog subpopulation disappears and only high-Nanog state remains. The green curves are the ODE trajectories of induced cell reprogramming path started from the ME differentiated state. The backgrounds are the potential landscapes after induction, showing the equilibrium distribution of high-Nanog / low-Nanog subpopulations when external inductions are turned on. **b**, **d**, **f** The equilibrium distribution of Oct4 and Nanog among simulated cell population (*N* = 10,000) after induction: (**b**) the fraction of low-Nanog subpopulation for mild induction is 47.3%; (**d**) the fraction of low-Nanog subpopulation for strong induction is up to 97.9%; (**f**) almost no low-Nanog subpopulation for strong induction of Oct4 and Sox2 coupled with additional Nanog activation

We next study the role of Nanog in the induction process by exploring four strategies previously studied in the “Seesaw” model of iPS reprogramming [33]. Specifically, the four strategies are: activating stemness genes Oct4 and Sox2; activating one stemness gene and another specifier gene group (Oct4 & ECT, or Sox2 & ME); activating one stemness gene while repressing another lineage specifier gene group (+Oct4 & -ME, or +Sox2 & -ECT); and equal activation of two lineage specifier genes ME and ECT. One important step among those strategies is the activation of Oct4 and Sox2, for which the feedbacks from Nanog to Oct4 and Sox2 provide additional regulations. When the activation strengths of Oct4 and Sox2 are mild (Fig. 4a & b), the high-Nanog cells dominate in the induction process. However, when the activation becomes stronger (Fig. 4c & d), the low-Nanog intermediate state dominates. This can be intuitively understood as a result of the repression of Oct4/Sox2 on Nanog: when Oct4 and Sox2 are highly induced, Nanog will be repressed to a low level, leading to more incomplete reprogrammed cells and slower reprogramming process. We note that introducing Nanog activation in the induction process could rescue reprogramming by removing the intermediate Nanog state (Fig. 4e & f). This observation is consistent with the stable pluripotent state with high-doxycycline in D8H in [44], where Oct4 and Nanog have been rapidly upregulated to steady level, and remain after lowering the doxycycline concentration. The same mechanism can explain the two similar strategies of reprogramming involving the direct activation of Oct4 or Sox2: activating Oct4 and repressing MEs (Additional file 7: Figure S6A-C), and activating both Sox2 and ECTs (Additional file 7: Figure S6D-F), where additional Nanog activation could remove the low-Nanog state. In the strategy of activating two lineage specifiers without Oct4 and Sox2, although the repression of Nanog due to Oct4/Sox2 over-activation may be less significant, additional Nanog activation may still benefit through removing the intermediate state (Additional file 7: Figure S6G & H).

Together, we suggest that additional Nanog activation can elevate Nanog level that is repressed by over-activated Oct4/Sox2 during induction, which can boost reprogramming efficiency by eliminating the Nanog intermediate state.

## Discussion

Nanog has been recognized as a critical pluripotency gene in ES cell differentiation and iPS cell reprogramming [26, 27]. While the low-Nanog state of stem cell has been speculated as a “gate-keeper” [25], the roles of the heterogeneous bimodal Nanog expression remain elusive. In this work, through analyzing a five-node gene regulatory network, including both stemness genes Oct4, Sox2, Nanog and specifier genes MEs and ECTs, we investigated the role of Nanog in stem cell differentiation and iPS cell reprogramming. In differentiation, the low-Nanog state is found to serve as an intermediate state in stem cell differentiation and to promote the differentiation process. On the other hand, the bimodal distribution of Nanog is found to have a negative effect on the iPS cell reprogramming. Additional Nanog activation is shown to prevent the reprogrammed iPS cell from trapping in the Nanog intermediate state due to over-activated Oct4/Sox2.

**Fig. 5:**
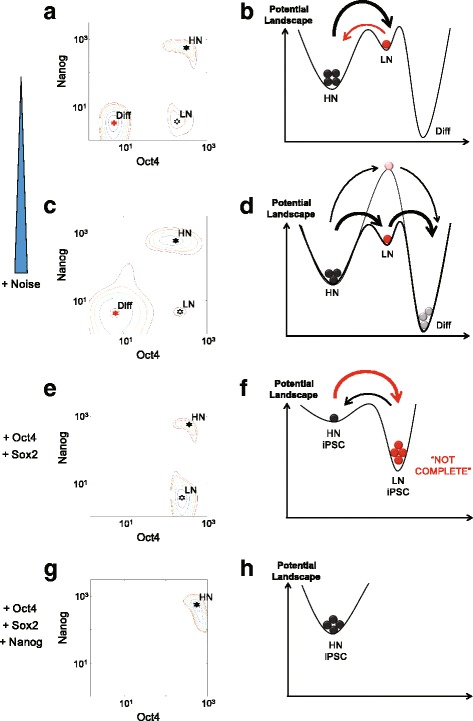
Summary of the dual roles of Nanog in differentiation and reprogramming. **a**, **c**, **e**, **g** Cellular states shown on the potential landscape; (**b**, **d**, **f**, **h**) Corresponding metaphoric energy curves. **a**, **b** Spontaneous transition between high/low-Nanog states in stem cells; **c**, **d** Differentiation tendency of stem cells: the low-Nanog intermediate state reduces the transition barrier in the differentiation process, making stem cell more prone to differentiate. The solid curve is with the Nanog dynamics and the dotted curve without; **e**, **f** Reprogramming with only Oct4 and Sox2 activation: large fraction of low-Nanog cells reduces the efficiency of reprogramming; **g**, **h** Reprogramming with additional Nanog activation: elimination of the low-Nanog subpopulation increases reprogramming efficiency

Through analyzing the Waddington landscape and calculating the minimum action path of differentiation, we suggest that the dynamic Nanog heterogeneity, induced by gene expression noise, can be utilized to maintain the stemness while, in the meantime, providing flexibility in cell fate decisions (Fig. 5). Differentiation drives cells in the high-Nanog state to overcome the barrier in the landscape. Once the cells arrive at the noise-induced Nanog intermediate state, they become more prone to differentiate. The two-step process by going through the intermediate state allows “easier” differentiation than the one-step process bypassing the intermediate state. Our model shows that the negative feedback between Oct4-Sox2 and Nanog is the key to introducing the intermediate state for cell differentiation. In principle, such intermediate state could be produced by other mechanisms. For instance, the histone modification in the chromatin region of Oct4 and Nanog is randomly re-established during each cycle of cell division and chromosome duplication [12]. Consequently, stochastic inheritance of histone modifications may also control gene on/off switching, leading to the cell-cell heterogeneity [45, 46] and potential intermediate states. Moreover, it will be interesting to develop a more comprehensive model to capture the fully dynamic processes of reprogramming by including the transcription factors (e.g. gene nodes of Klf4, Myc [47]) and specified ME/ECT lineage specifiers.

Recently, there is some controversial discussion that the genetic reporter for Nanog may cause a bifurcation in the underlying dynamics that leads to heterogeneous Nanog expression induced by the bistability [48]. Such problem of measurement in cells could induce the Nanog fluctuations. However, gene reporter strategy is generic, and may also cause the similar measurement problem of the other pluripotent genes such as Oct4 and Sox2 whose heterogeneity has not been observed in the literatures. Moreover, our present model shows the negative feedback between Nanog and Oct4-Sox2 can constitute an excitable system, leading to a recurring stochastic transition between high and low Nanog states of pluripotent cells and the bimodal distribution of Nanog. Thus, the bistability required in [48] is not necessarily needed for the heterogeneous Nanog expression. Furthermore, perturbation of Nanog dynamics by the fluorenscent reporter system may not full account for the heterogeneity of Nanog expression under all culture conditions. The datasets using single cell RNA-Seq can support the bimodal Nanog expression for mouse embryonic stem cells cultured in serum and LIF [4, 35], where the genetic reporter is excluded.

As a strategy in regulating stem cell properties, multiple intermediate states have been discovered in epithelial-mesenchymal transition (EMT) process to control cellular plasticity [49–51]. Similar to microRNA and Ovol2 in the EMT regulatory network, which is critical to inducing one or more intermediate (“hybrid”) states, Nanog is a key factor in the late stage of reprogramming process to promote the incomplete reprogrammed cells to attain the full pluripotent state. Identification of Nanog-related external signals and genes will help to improve strategies for iPS cell reprogramming.

## Conclusions

In summary, our modeling work has identified novel roles of Nanog in stem cell regulation. Such approach can include more biological details or other stem cell markers for more in-depth exploration. Our work offers a general method for analyzing key regulatory factors controlling cell differentiation and reprogramming.

## Methods

### Stochastic differential equations and parameters of the five-node model

The stochastic differential equations and parameters of the five-node model are listed below, where [*O*], [*S*], [*N*], [*M*] and [*E*] represent the expression level of Oct4, Sox2, Nanog, MEs and ECTs respectively. The parameters of the model are listed in Additional file 1: Table S1.

1.1 $$ \frac{\mathrm{d}\left[\mathrm{O}\right]}{\mathrm{d}\mathrm{t}}=\frac{1}{\tau_p}\left[\left({\beta}_{p\_ KM}+{\beta}_p\bullet \frac{\left[O\right]\left[S\right]}{\left[O\right]\left[S\right]+{K}_{p_{\_ act}}^2}\bullet \frac{\left[N\right]}{\left[N\right]+{K}_{p_{\_ Na}}}\right)\bullet \frac{K_{p_{\_ inh}}^{10}}{K_{p{\_}_{inh}}^{10}+{\left[M\right]}^{10}}\bullet \frac{K_{p{\_}_{inh}}^{10}}{K_{p{\_}_{inh}}^{10}+{\left[E\right]}^{10}}+{\beta}_{p0}-{d}_O\left[O\right]\right]+{\sigma}_O\left[O\right]\bullet \xi (t) $$

1.2 $$ \frac{\mathrm{d}\left[\mathrm{S}\right]}{\mathrm{d}\mathrm{t}}=\frac{1}{\tau_p}\left[\left({\beta}_{p\_ KM}+{\beta}_p\bullet \frac{\left[O\right]\left[S\right]}{\left[O\right]\left[S\right]+{K}_{p_{\_ act}}^2}\bullet \frac{\left[N\right]}{\left[N\right]+{K}_{p_{\_ Na}}}\right)\bullet \frac{K_{p_{\_ inh}}^{10}}{K_{p{\_}_{inh}}^{10}+{\left[M\right]}^{10}}\bullet \frac{K_{p{\_}_{inh}}^{10}}{K_{p{\_}_{inh}}^{10}+{\left[E\right]}^{10}}+{\beta}_{p0}-{d}_S\left[S\right]\right]+{\sigma}_S\left[S\right]\bullet \xi (t) $$

1.3 $$ \frac{\mathrm{d}\left[\mathrm{N}\right]}{\mathrm{d}\mathrm{t}}={\beta}_{Na\_ OS}+\left({\beta}_{Na}\bullet \frac{{\left[N\right]}^2}{K_{Na\_ act}^2+{\left[N\right]}^2+{S}_{Na\_ OS}\left[O\right]\left[S\right]}\right)\bullet \frac{K_{p\_ inh}^{10}}{K_{p\_ inh}^{10}+{\left[M\right]}^{10}}\bullet \frac{K_{p\_ inh}^{10}}{K_{p\_ inh}^{10}+{\left[E\right]}^{10}}-{d}_N\left[N\right]+\left({\sigma}_N\left[O\right]+{\sigma}_{N_0}\right)\bullet \xi (t) $$

1.4 $$ \frac{\mathrm{d}\left[\mathrm{M}\right]}{\mathrm{d}\mathrm{t}}=\frac{1}{\tau_D}\left\{{\beta}_d\left[w\bullet \frac{{\left[O\right]}^9}{{\left[O\right]}^9+{K}_a^9}+\frac{K_i^{10}}{K_i^{10}+{\left[S\right]}^{10}}\bullet \frac{K_{d\_ inh}^{10}}{K_{d\_ inh}^{10}+{\left[E\right]}^{10}}\right]+{\beta}_{d0}-{d}_M\left[M\right]\right\}+{\sigma}_M\left[M\right]\bullet \xi (t) $$

1.5 $$ \frac{\mathrm{d}\left[\mathrm{E}\right]}{\mathrm{d}\mathrm{t}}=\frac{1}{\tau_D}\left\{{\beta}_d\left[w\bullet \frac{{\left[S\right]}^9}{{\left[S\right]}^9+{K}_a^9}+\frac{K_i^{10}}{K_i^{10}+{\left[O\right]}^{10}}\bullet \frac{K_{d\_ inh}^{10}}{K_{d\_ inh}^{10}+{\left[M\right]}^{10}}\right]+{\beta}_{d0}-{d}_E\left[E\right]\right\}+{\sigma}_E\left[E\right]\bullet \xi (t) $$

The Oct4-Sox2 protein complex will activate Nanog expression more sensitively, i.e. with a low critical value in the activation Hill function. While at the same time, Oct4-Sox2 complex will repress Nanog with a high critical value in the repression Hill function. As the activation strength of Oct4-Sox2 to Nanog (parameter *β*_*Na*_*OS*_ in the model) is much smaller than that of Nanog self-activation (*β*_*Na*_), the Hill-activation term is simplified as a constant term (*β*_*Na*_*OS*_) in the model. Finally, both MEs and ECTs repress the expression of Nanog. The downstream interactions of Nanog to lineage specifier genes are complicated, which are not considered in this model for simplicity. The noise in the system is added in the form of *σ*_*X*_[*X*] ∙ *ξ*(*t*), where *σ*_*X*_ indicates the noise amplitude of each gene, and *ξ*(*t*) is the Gaussian white noise with zero mean and unit variance.

The setting of parameter values refers to the previous modeling works [2, 3], shown in Additional file 2: Figure S1A. Note that the copy numbers of Oct4 and Nanog in the model are adjusted to fit the results of mice E14IVc ES cell line cultured in serum and LIF [2], which can be adjusted and scaled for other stem cell lines. Sox2 is assumed to be symmetric to Oct4, so most parameters for Oct4 and Sox2 are set to be identical. Thus, the expression levels of both Oct4 and Sox2 are high in pluripotent cell states and low in differentiated cell states. If there is a strong initial imbalance of Oct4 / Sox2, such as high Oct4 and low Sox2, the system will lead to an imbalanced expression of lineage specifiers: high MEs and low ECTs. The high expression of MEs then represses Oct4, leading to differentiation toward the ME state, where both Oct4 and Sox2 are low in expression. On the other hand, if the initial imbalance of Oct4/Sox2 is lower than a threshold, which cannot trigger the differentiation process, the cell then returns to the pluripotent steady state, where both Oct4 and Sox2 are highly expressed. As a result, the imbalanced expression of Oct4 and Sox2 could only be a transient state, and such imbalance disappears after the cells either commit to differentiation or remain in the pluripotent state. The noise levels of each gene are adjusted to fit the previous experimental results of mice E14IVc ES cell line [2]. Note that the degradation rate 1 a.u.^− 1^ of the arbitrary timescale in the model is corresponding to 3 × 10^−5^ s^− 1^ under the real timescale. For the re-establishment time (Fig. 2b) and dwell time distribution (Fig. 2c), the timescale of the system is scaled from a.u. to day.

### Simplified two-dimensional Oct4-Nanog model

The regulatory relationships between Oct4 and Nanog are extract out of the five-node model (where the Sox2 is assumed totally symmetric to Oct4), and the concentration of MEs and ECTs are set up as the constant level under the pluripotent state. The stochastic differential equations are displayed below, where [*O*], [*N*] represent the level of Oct4 and Nanog respectively, and the parameters of the model are listed in Table S2. The behavior of excitable system can be illustrated on the two-dimensional phase plane in terms of nullclines and vector fields (Additional file 3: Figure S2).

2.1 $$ \frac{\mathrm{d}\left[\mathrm{O}\right]}{\mathrm{d}\mathrm{t}}=\frac{1}{\tau_p}\left[{\beta}_{O\_ KM}+{\beta}_O\bullet \frac{{\left[O\right]}^2}{{\left[O\right]}^2+{K}_{p_{\_ act}}^2}\bullet \frac{\left[N\right]}{\left[N\right]+{K}_{O\_ Na}}-{d}_O\left[O\right]\right]+{\sigma}_O\left[O\right]\bullet \xi (t) $$

2.2 $$ \frac{\mathrm{d}\left[\mathrm{N}\right]}{\mathrm{d}\mathrm{t}}=\left[{\beta}_{Na\_ OS}+{\beta}_{Na}\bullet \frac{{\left[N\right]}^2}{K_{Na\_ act}^2+{\left[N\right]}^2+{S}_{Na\_ OS}{\left[O\right]}^2}-{d}_N\left[N\right]\right]+\left({\sigma}_N\left[O\right]+{\sigma}_{N\_0}\right)\bullet \xi (t) $$

### Parameter sensitivity analysis of the five-node model

To test the robustness of the model, each parameter is individually increased/decreased by 20%, and the relative changes of the low-Nanog distribution ratio (in the time course, blue bar), the average value of Oct4 expression level (green bar), and the average value of Nanog expression level within the high-Nanog state (red bar) is illustrated in Additional file 8: Figure S3. The parameters of the Oct4/Sox2 expression terms are more sensitive (the first panel from the left), and the parameters of the expression terms of Nanog, MEs and ECTs are more robust (the second, third panel from the left).

### The control model excluding the dynamics of Nanog for MAM analysis

To verify the role of Nanog during the differentiation process, we set up a control model, where the dynamics and the regulatory relationships of Nanog are removed from the five-node model. As the activation terms of Nanog to Oct4 and Sox2 ($\frac{\left[N\right]}{\left[N\right]+{K}_{p\_ Na}}$ in the formulas 1.1 and 1.2) are the only input regulations from Nanog to the rest part of the network, the concentration value of Nanog in those two terms is set as the constant value of highly expressed steady state value of Nanog, so that the steady state values of the other four genes can remain unchanged at the same time.

### The model with external induction input terms

In order to analyze the induced iPS reprogramming process, some constant input terms are added into the model. The input parameters for gene expression activation (*C*_*O*_, *C*_*S*_, *C*_*N*_, *C*_*M*_, *C*_*E*_) indicate the extra, constant protein production rates by exogeneous expression (by virus vector, for example) for each gene, which are set as 0 without any inductions. Note that the strengths of external induction terms are scaled (*K*_*O*_ = *K*_*S*_ = *K*_*N*_ = *K*_*M*_ = *K*_*E*_ = 400). While the gene expression repressions due to specific miRNA molecules silencing the translation of certain mRNA molecules are modeled by the multiplier terms before the entire regulated expression rate terms for each gene (*I*_*O*_, *I*_*S*_, *I*_*N*_, *I*_*M*_, *I*_*E*_), which is set as 1 without any inductions. All other parameters remained unchanged.

3.1 $$ \frac{\mathrm{d}\left[\mathrm{O}\right]}{\mathrm{d}\mathrm{t}}=\frac{1}{\tau_p}\left[{I}_O\bullet \left[\left({\beta}_{p\_ KM}+{\beta}_p\bullet \frac{\left[O\right]\left[S\right]}{\left[O\right]\left[S\right]+{K}_{p\_ act}^2}\bullet \frac{\left[N\right]}{\left[N\right]+{K}_{p\_ Na}}\right)\bullet \frac{K_{p\_ inh}^{10}}{K_{p\_ inh}^{10}+{\left[M\right]}^{10}}\bullet \frac{K_{p\_ inh}^{10}}{K_{p\_ inh}^{10}+{\left[E\right]}^{10}}+{C}_O\bullet {K}_O\right]+{\beta}_{p0}-{d}_O\left[O\right]\right]+{\sigma}_O\left[O\right]\bullet \xi (t) $$

3.2 $$ \frac{\mathrm{d}\left[\mathrm{S}\right]}{\mathrm{d}\mathrm{t}}=\frac{1}{\tau_p}\left[{I}_S\bullet \left[\left({\beta}_{p\_ KM}+{\beta}_p\bullet \frac{\left[O\right]\left[S\right]}{\left[O\right]\left[S\right]+{K}_{p\_ act}^2}\bullet \frac{\left[N\right]}{\left[N\right]+{K}_{p\_ Na}}\right)\bullet \frac{K_{p\_ inh}^{10}}{K_{p\_ inh}^{10}+{\left[M\right]}^{10}}\bullet \frac{K_{p\_ inh}^{10}}{K_{p\_ inh}^{10}+{\left[E\right]}^{10}}+{C}_S\bullet {K}_S\right]+{\beta}_{p0}-{d}_S\left[S\right]\right]+{\sigma}_S\left[S\right]\bullet \xi (t) $$

3.3 $$ \frac{\mathrm{d}\left[\mathrm{N}\right]}{\mathrm{d}\mathrm{t}}={I}_N\left[{\beta}_{Na\_ OS}+\left({\beta}_{Na}\bullet \frac{{\left[N\right]}^2}{K_{Na\_ act}^2+{\left[N\right]}^2+{S}_{Na\_ OS}\left[O\right]\left[S\right]}\right)\bullet \frac{K_{p\_ inh}^{10}}{K_{p\_ inh}^{10}+{\left[M\right]}^{10}}\bullet \frac{K_{p\_ inh}^{10}}{K_{p\_ inh}^{10}+{\left[E\right]}^{10}}+{C}_N\bullet {K}_N\right]-{d}_N\left[N\right]+\left({\sigma}_N\left[O\right]+{\sigma}_{N_0}\right)\bullet \xi (t) $$

3.4 $$ \frac{\mathrm{d}\left[\mathrm{M}\right]}{\mathrm{d}\mathrm{t}}=\frac{1}{\tau_D}\left\{{I}_M\left[{\beta}_d\bullet \left[w\bullet \frac{{\left[O\right]}^9}{{\left[O\right]}^9+{K}_a^9}+\frac{K_i^{10}}{K_i^{10}+{\left[S\right]}^{10}}\bullet \frac{K_{d_{inh}}^{10}}{K_{d_{inh}}^{10}+{\left[E\right]}^{10}}\right]+{C}_M\bullet {K}_M+{\beta}_{d0}\right]-{d}_M\left[M\right]\right\}+{\sigma}_M\left[M\right]\bullet \xi (t) $$

3.5 $$ \frac{\mathrm{d}\left[\mathrm{E}\right]}{\mathrm{d}\mathrm{t}}=\frac{1}{\tau_D}\left\{{I}_E\left[{\beta}_d\bullet \left[w\bullet \frac{{\left[S\right]}^9}{{\left[S\right]}^9+{K}_a^9}+\frac{K_i^{10}}{K_i^{10}+{\left[O\right]}^{10}}\bullet \frac{K_{d_{inh}}^{10}}{K_{d_{inh}}^{10}+{\left[M\right]}^{10}}\right]+{C}_E\bullet {K}_E+{\beta}_{d0}\right]-{d}_E\left[E\right]\right\}+{\sigma}_E\left[E\right]\bullet \xi (t) $$

### The sampling method of the potential landscape

The landscape functions in the non-equilibrium systems have been widely used to model the gene switching and cell fate transition [52, 53], which serves as an analogy of the energy function in the equilibrium systems. However, several different landscape functions have been proposed by biophysicists and applied mathematicians [38–40]. The mathematical relations and differences between different landscapes are elucidated in [54]. Here we choose the simplest one, using the negative logarithm of the probability distribution in the phase space under the steady state,− log(*P*_*ss*_), as the definition of the potential landscape. Note that the gradient of − log(*P*_*ss*_) is no longer corresponding to the vector field direction of the dynamical system. And the landscape of− log(*P*_*ss*_) is actually, to some extent, limited as an intuitive sketch of cellular state distribution in the phase space. The probability distributions (*P*_*ss*_) in the phase space of the figures above are produced by simulating the system for more than 10^7^ time a.u., and the probability density of the trajectories are projected into the three-dimensional space, with the x, y, z axis as $\sqrt{\left[ Oct4\right]\bullet \left[ Sox2\right]}$, [*Nanog*] and [*MEs*] − [*ECTs*] (e.g. Fig. 1b); or two-dimensional space in other figures, with the x, y axis as $\sqrt{\left[ Oct4\right]\bullet \left[ Sox2\right]}$ and [*Nanog*] (e.g. Fig. 1c). The color scale of the potential landscape measures the energy value, indicating the probability density for the cell state to appear in that certain region.

### The method of minimum action path

The Wentzell-Freidlin theory of large deviation gives an estimate of the probability of the paths in terms of an action functional. A key result of this theory is that the most probable path minimizes the action functional associated with the random dynamical system, i.e., the most probable path is the Minimum Action Path.

In order to find the MAP between two steady states, we follow the minimum action method in [42] to compute the numerical solutions with the time interval [0, 100]. We apply the BFGS algorithm for numerical optimization.

## Additional files

Additional file 1: **Table S1.** Parameters used in Eq. (1) for the five-node model. (DOCX 50 kb)

Additional file 2: **Figure S1.** Typical temporal trajectories of stochastic gene expressions at the ME differentiated cell state. ME state is a stable state, and the noise-driven transition from differentiated states (low Oct4, Sox2 and Nanog) to pluripotent states (high Oct4 and Sox2, low MEs and ECTs) cannot occur spontaneously. (TIFF 1916 kb)

Additional file 3: **Figure S2.** The simplified two-dimensional Oct4-Nanog model on the phase plate and the distribution of Oct4. (A)The nullclines and the vector field of the simplified two-dimensional Oct4-Nanog model on the phase plate. A typical trajectory is illustrated to indicate the excitable mechanism of the model. (d[*Oct*4]/d*t* = 0: Red line; d[*Nanog*]/d*t* = 0: Blue line.) (B) Distributions of Sox2 level within simulated cell population (*N* = 10,000). (PDF 102 kb)

Additional file 4: **Table S2.** Parameters used in Eq. (2) for the simplified Oct4-Nanog model. (DOCX 42 kb)

Additional file 5: **Figure S4.** The MAPs of the differentiation process with two different initial paths in the WT model. The MAPs (white curves) starting from the pluripotent state (the green point) to the ME differentiated state (the blue point) are insensitive to different initial conditions (purple curves): (A) a smooth curve passing by the low-Nanog state; (B) a smooth curve far from low-Nanog state. (PDF 614 kb)

Additional file 6: **Figure S5.** The MAP of the reprogramming process in the WT model. The MAP (white curve) starting from the ME differentiated state (the blue point) to the pluripotent state (the green point) is different from that of differentiation process (Fig. 3A). The green dotted line is the ODE trajectory to compare with the MAP. (PDF 3338 kb)

Additional file 7: **Figure S6.** Three different strategies of reprogramming demonstrate additional Nanog activation is necessary to maintain the high Nanog level and promote the efficient cell reprogramming. (A-C) Strategy by of activating Oct4 and repressing MEs. (A) *C*_0_ = *I*_*m*_ = 0.3; (B) *C*_0_ = *I*_*m*_ = 0.5; (C) *C*_0_ = *I*_*m*_ = *C*_*n*_ = 0.5; (D-F) Strategy of activating Sox2 and ECTs. (D) *C*_*m*_ = 0.3, *C*_*s*_ = 0.06; (E) *C*_*m*_ = 0.5, *C*_*S*_ = 0.1; (F) *C*_*m*_ = 0.5, *C*_*S*_ = 0.1, *C*_*n*_ = 0.5; (G-H) Strategy of activating MEs and ECTs. (G) *C*_*m*_ = *C*_*e*_ = 0.3; (H) *C*_*m*_ = *C*_*e*_ = *C*_*n*_ = 0.3. (PDF 2322 kb)

Additional file 8: **Figure S3.** Parameter sensitivity analysis for the model. Illustration of the relative changes of the low-Nanog distribution ratio (blue bar), the average Oct4 level (green bar), and the average Nanog level of high-Nanog population (red bar). (TIFF 699 kb)

## Abbreviations

ECTs: Ectoderm genes
ES: Embryonic stem
iPS: Induced pluripotent stem
MAP: Minimum Action Path
MEs: Mesendoderm genes
WT: Wild-type
