# Supplementary material for: Nanog induced intermediate state in regulating stem cell differentiation and reprogramming
Source: BMC Syst Biol. 2018 Feb 27;12:22. doi: 10.1186/s12918-018-0552-3 (PMC6389130; doi:10.1186/s12918-018-0552-3)
Supplement: Supplementary file 1 — Table S1. Parameters used in Eq. (1) for the five-node model. (DOCX 50 kb) [file 12918_2018_552_MOESM1_ESM.docx]

**Supplementary Table 1.**

| Parameter | Value | Parameter | Value | Parameter | Value |
| --- | --- | --- | --- | --- | --- |
| $\beta_{p\_KM}$ | 100 | $K_{p\_act}$ | 372.5 | $d_{O}=d_{s}=d_{N}=d_{M}=d_{E}$ | 1 |
| $\beta_{p}$ | 1400 | $K_{p\_Na}$ | 750 | $\tau_{p}$ | 20 |
| $\beta_{p^{0}}$ | 2.5 | $K_{p\_inh}$ | 225 | $\tau_{D}$ | 4 |
| $\beta_{Na\_OS}$ | 4.5 | $K_{Na\_act}$ | 48 | $w$ | 0.03 |
| $\beta_{NA}$ | 680 | $S_{Na\_OS}$ | 0.675 | $\sigma_{O}=\sigma_{S}$ | 0.1 |
| $\beta_{d}$ | 340 | $K_{a}$ | 450 | $\sigma_{N}$ | 0.2 |
| $\beta_{d^{0}}$ | 0.81 | $K_{i}$ | 45 | $\sigma_{N_{0}}$ | 2 |
|  |  | $K_{d\_inh}$ | 100 | $\sigma_{M}=\sigma_{E}$ | 0.05 |

Table S1. Parameters used in Eq. (1) for the five-node model.
