# Supplementary figures and images for: Nanog induced intermediate state in regulating stem cell differentiation and reprogramming
Source: BMC Syst Biol. 2018 Feb 27;12:22. doi: 10.1186/s12918-018-0552-3 (PMC6389130; doi:10.1186/s12918-018-0552-3)

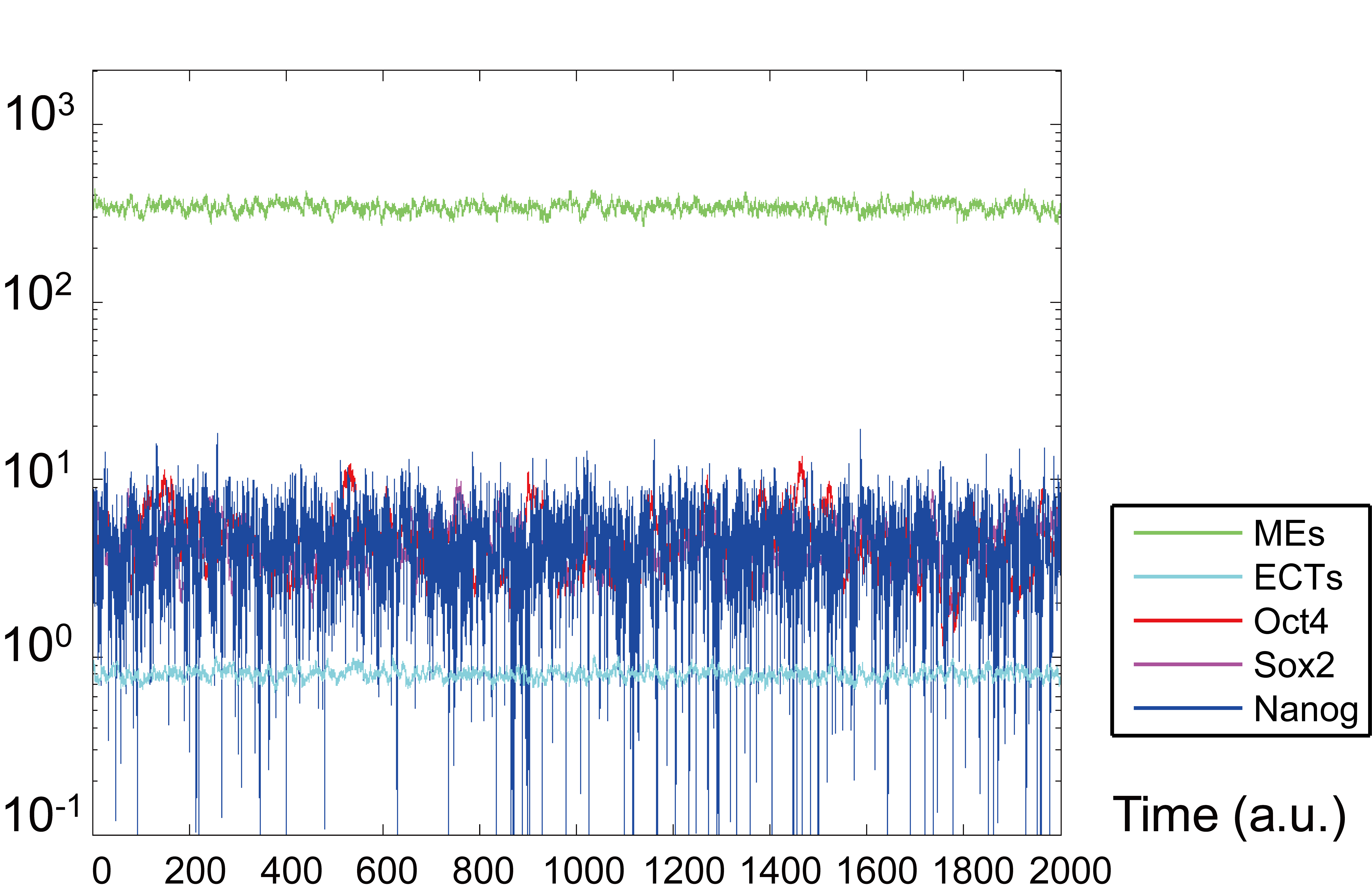

Supplement: Supplementary file 2 — Figure S1. Typical temporal trajectories of stochastic gene expressions at the ME differentiated cell state. ME state is a stable state, and the noise-driven transition from differentiated states (low Oct4, Sox2 and Nanog) to pluripotent states (high Oct4 and Sox2, low MEs and ECTs) cannot occur spontaneously. (TIFF 1916 kb) [file 12918_2018_552_MOESM2_ESM.tif]

**A**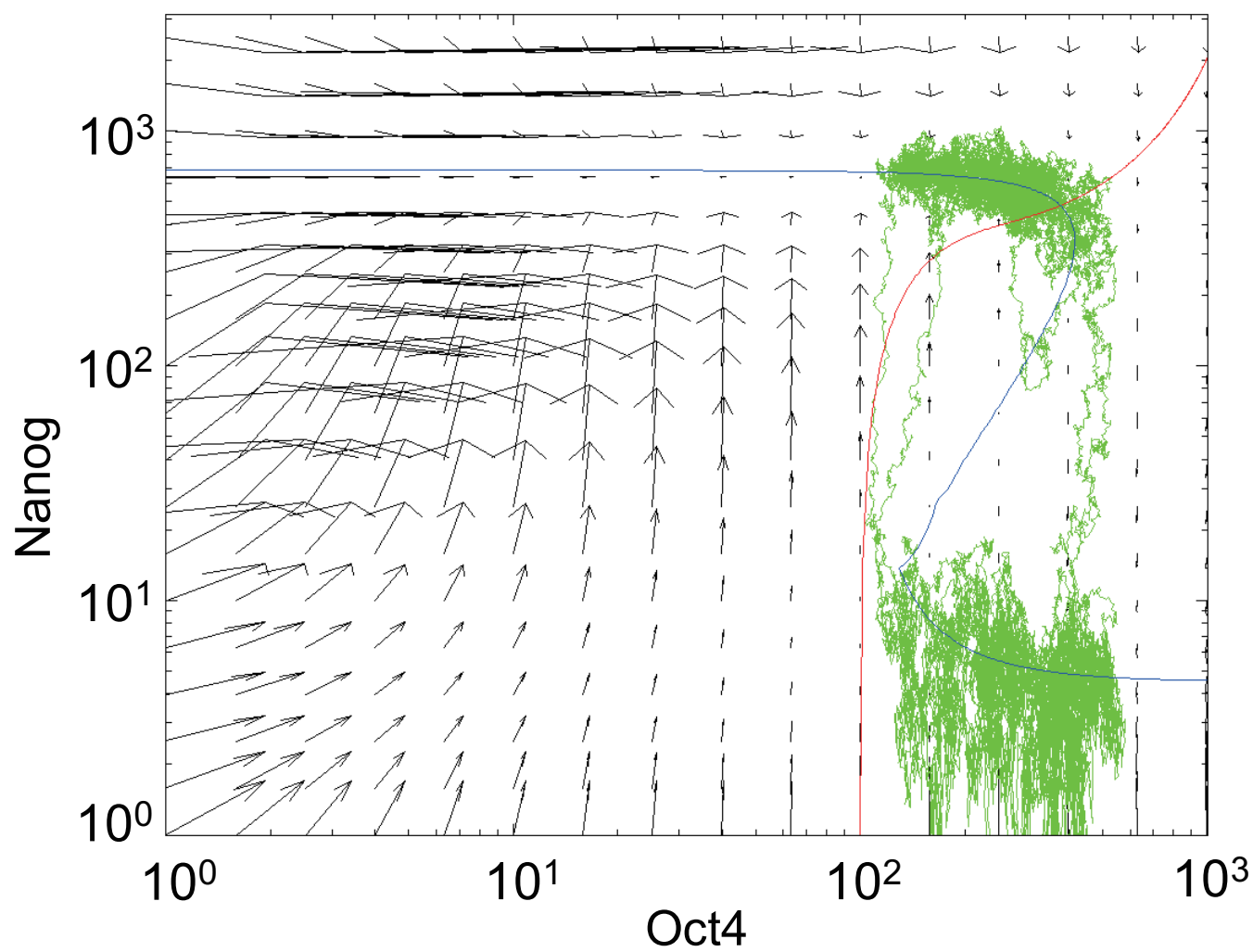**B**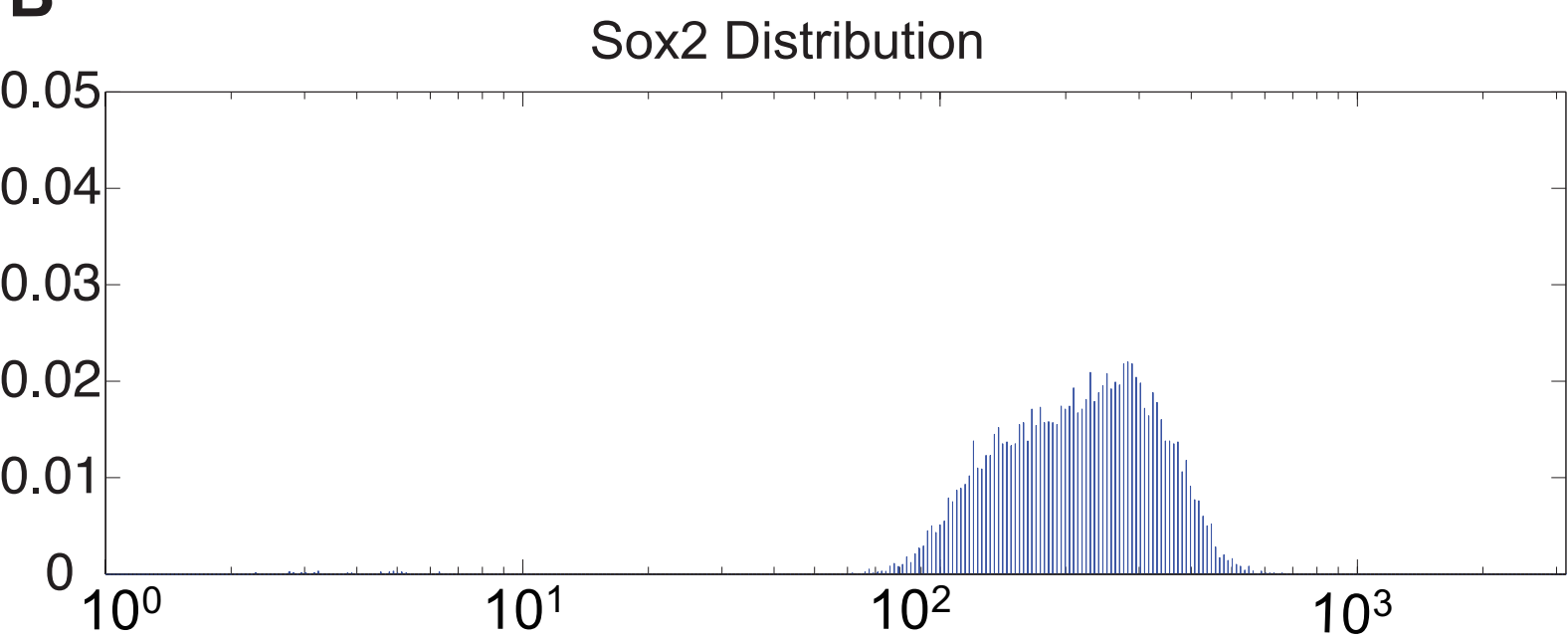

Supplement: Supplementary file 3 — Figure S2. The simplified two-dimensional Oct4-Nanog model on the phase plate and the distribution of Oct4. (A)The nullclines and the vector field of the simplified two-dimensional Oct4-Nanog model on the phase plate. A typical trajectory is illustrated to indicate the excitable mechanism of the model. (d[Oct4]/dt = 0: Red line; d[Nanog]/dt = 0: Blue line.) (B) Distributions of Sox2 level within simulated cell population (N = 10,000). (PDF 102 kb) [file 12918_2018_552_MOESM3_ESM.pdf]

**A**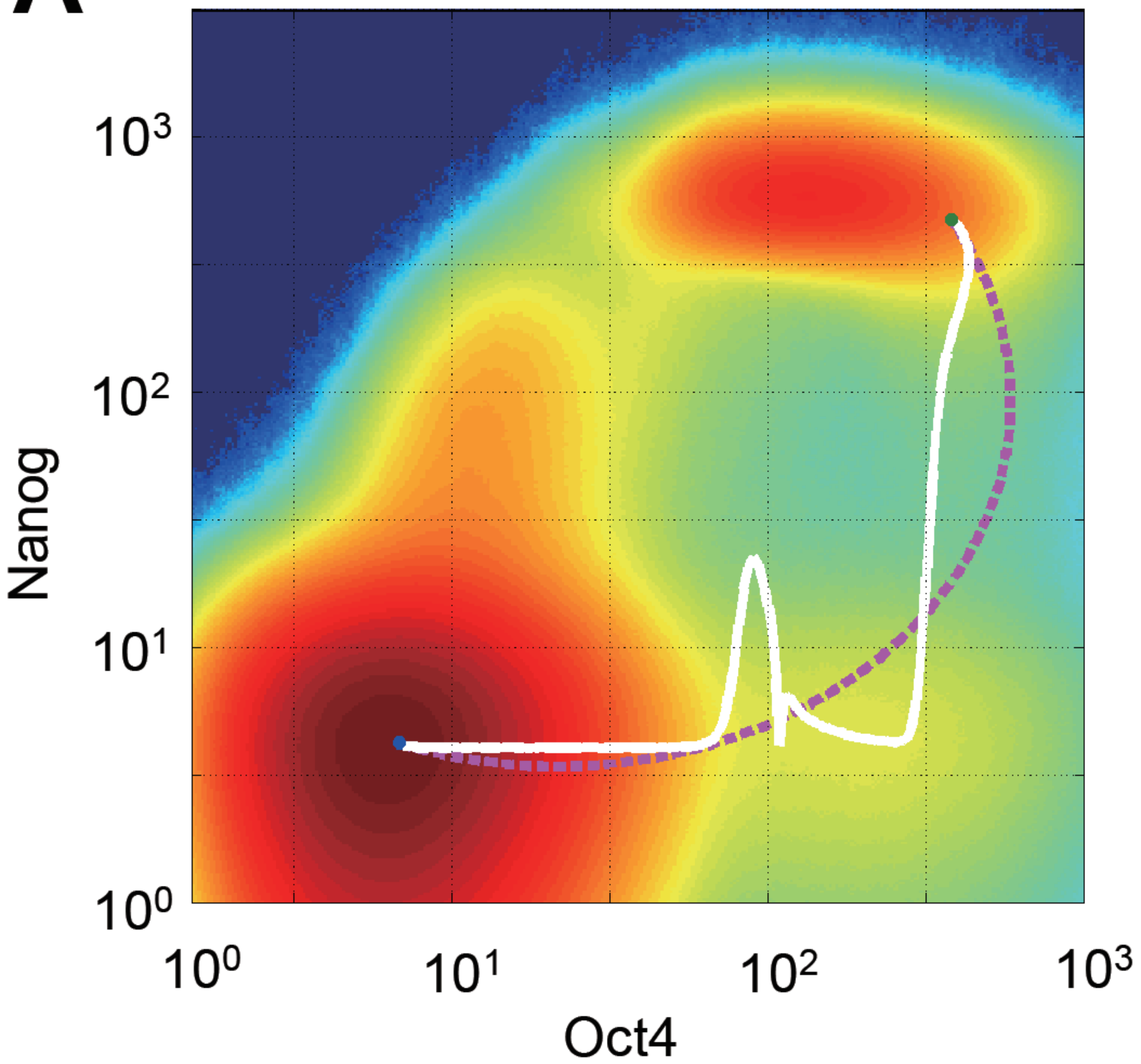**B**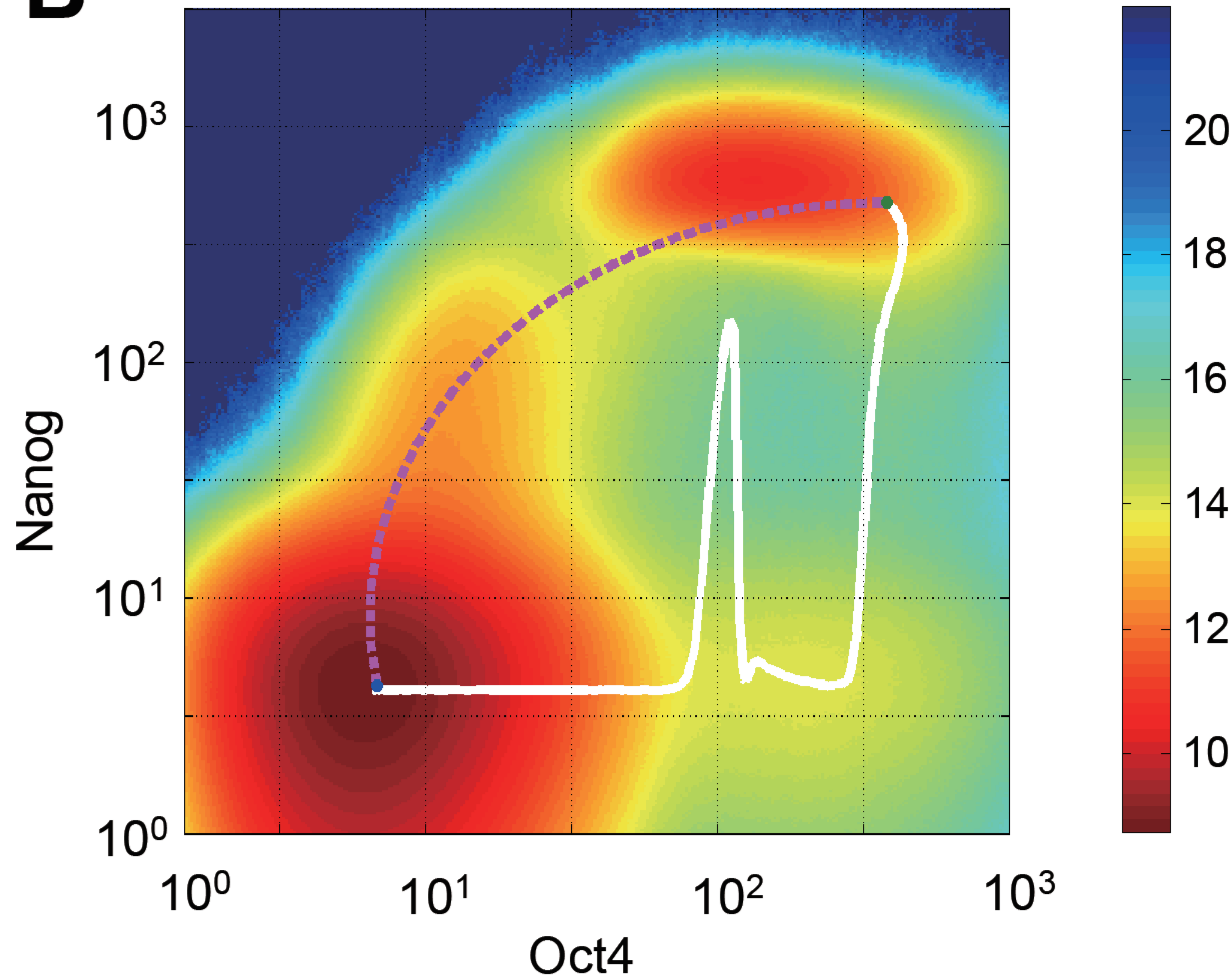

Supplement: Supplementary file 5 — Figure S4. The MAPs of the differentiation process with two different initial paths in the WT model. The MAPs (white curves) starting from the pluripotent state (the green point) to the ME differentiated state (the blue point) are insensitive to different initial conditions (purple curves): (A) a smooth curve passing by the low-Nanog state; (B) a smooth curve far from low-Nanog state. (PDF 614 kb) [file 12918_2018_552_MOESM5_ESM.pdf]

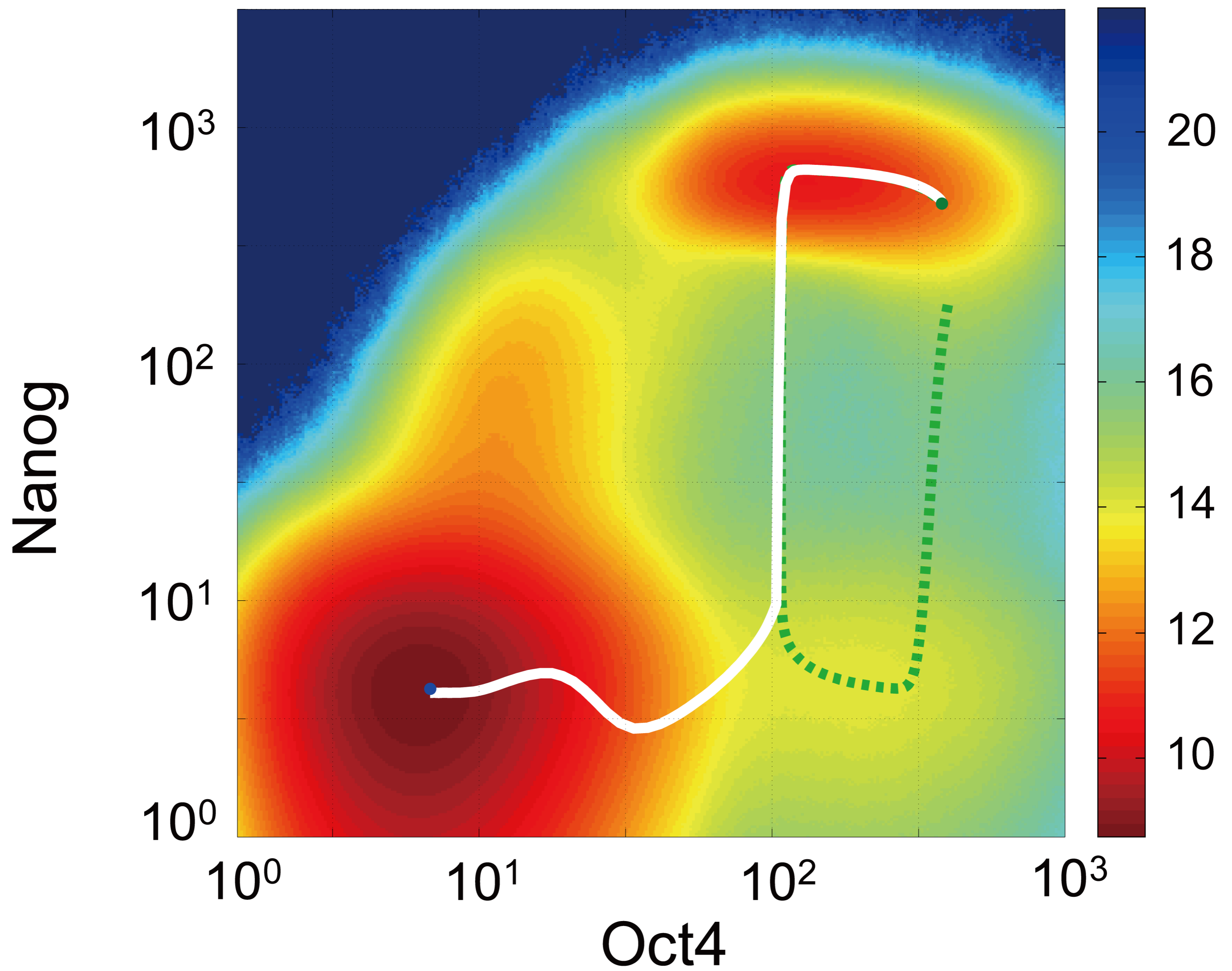

Supplement: Supplementary file 6 — Figure S5. The MAP of the reprogramming process in the WT model. The MAP (white curve) starting from the ME differentiated state (the blue point) to the pluripotent state (the green point) is different from that of differentiation process (Fig. 3A). The green dotted line is the ODE trajectory to compare with the MAP. (PDF 3338 kb) [file 12918_2018_552_MOESM6_ESM.pdf]

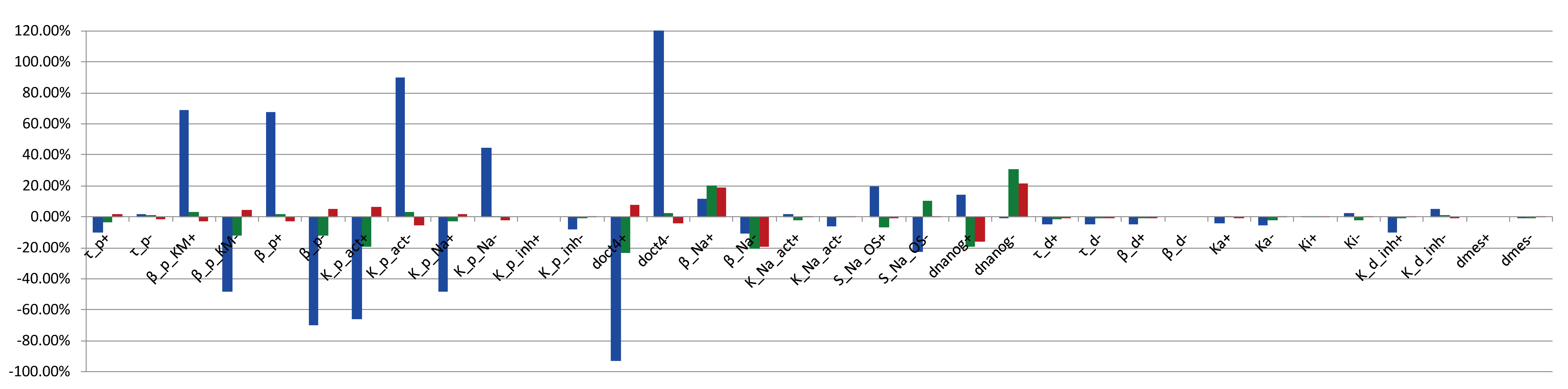

Supplement: Supplementary file 8 — Figure S3. Parameter sensitivity analysis for the model. Illustration of the relative changes of the low-Nanog distribution ratio (blue bar), the average Oct4 level (green bar), and the average Nanog level of high-Nanog population (red bar). (TIFF 699 kb) [file 12918_2018_552_MOESM8_ESM.tif]
