# Supplementary material for: Nanog induced intermediate state in regulating stem cell differentiation and reprogramming
Source: BMC Syst Biol. 2018 Feb 27;12:22. doi: 10.1186/s12918-018-0552-3 (PMC6389130; doi:10.1186/s12918-018-0552-3)
Supplement: Supplementary file 4 — Table S2. Parameters used in Eq. (2) for the simplified Oct4-Nanog model. (DOCX 42 kb) [file 12918_2018_552_MOESM4_ESM.docx]

**Supplementary Table 2**

| Parameter | Value | Parameter | Value | Parameter | Value |
| --- | --- | --- | --- | --- | --- |
| $\beta_{O\_KM}$ | 100 | $K_{p\_act}$ | 372.5 | $d_{O}=d_{N}$ | 1 |
| $\beta_{O}$ | 1400 | $K_{O\_Na}$ | 750 | $\tau_{p}$ | 20 |
| $\beta_{Na\_OS}$ | 4.5 | $K_{Na\_act}$ | 48 | $\sigma_{O}$ | 0.1 |
| $\beta_{Na}$ | 680 | $S_{Na\_OS}$ | 0.675 | $\sigma_{N}$ | 0.2 |
|  |  |  |  | $\sigma_{N_{0}}$ | 2 |

Table S2. Parameters used in Eq. (2) for the simplified Oct4-Nanog model.
