# Supplementary material for: Nanog induced intermediate state in regulating stem cell differentiation and reprogramming
Source: BMC Syst Biol. 2018 Feb 27;12:22. doi: 10.1186/s12918-018-0552-3 (PMC6389130; doi:10.1186/s12918-018-0552-3)
Supplement: Supplementary file 7 — Figure S6. Three different strategies of reprogramming demonstrate additional Nanog activation is necessary to maintain the high Nanog level and promote the efficient cell reprogramming. (A-C) Strategy by of activating Oct4 and repressing MEs. (A) C0 = Im = 0.3; (B) C0 = Im = 0.5; (C) C0 = Im = Cn = 0.5; (D-F) Strategy of activating Sox2 and ECTs. (D) Cm = 0.3, Cs = 0.06; (E) Cm = 0.5, CS = 0.1; (F) Cm = 0.5, CS = 0.1, Cn = 0.5; (G-H) Strategy of activating MEs and ECTs. (G) Cm = Ce = 0.3; (H) Cm = Ce = Cn = 0.3. (PDF 2322 kb) [file 12918_2018_552_MOESM7_ESM.pdf]

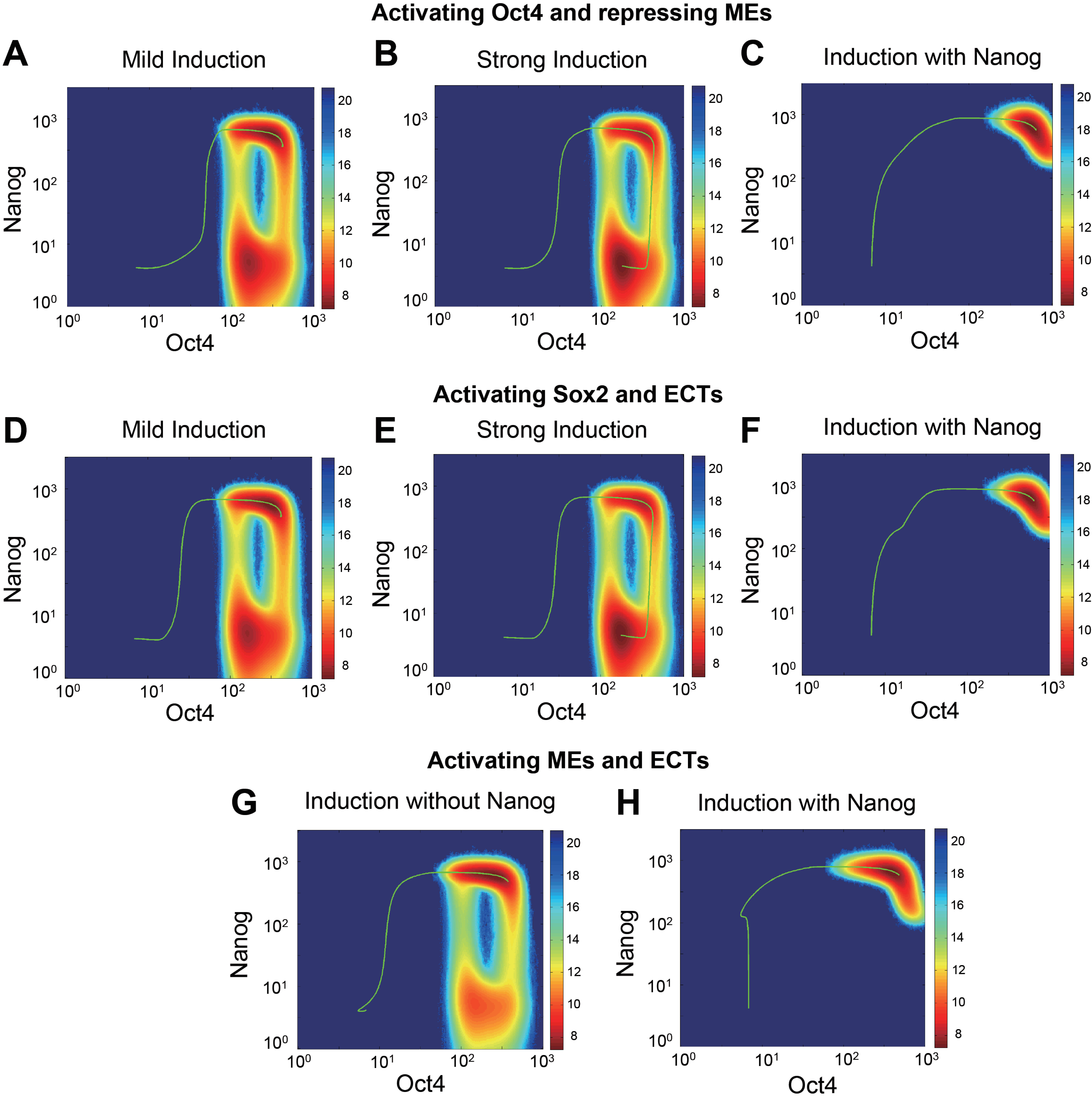

Activating Oct4 and repressing MEs
A
Mild Induction
B
Strong Induction
C
Induction with Nanog
Activating Sox2 and ECTs
D
Mild Induction
E
Strong Induction
F
Induction with Nanog
Activating MEs and ECTs
G
Induction without Nanog
H
Induction with Nanog
Nanog
Oct4
10^0
10^1
10^2
10^3
8
10
12
14
16
18
20
